# Mode of killing determines the necrotrophic response of oral bacteria

**DOI:** 10.1080/20002297.2023.2184930

**Published:** 2023-03-06

**Authors:** Naiera Zayed, Joana Figueiredo, Wannes Van Holm, Nico Boon, Kristel Bernaerts, Wim Teughels

**Affiliations:** a Department of Oral Health Sciences, University of Leuven (KU Leuven), Leuven, Belgium; b Center for Microbial Ecology and Technology (CMET), Ghent University (UGent), Gent, Belgium; c Faculty of Pharmacy, Menoufia University, Shebin El-Kom, Egypt; d Faculty of bioscience engineering, University of Minho, Braga, Portugal; e Chemical and Biochemical Reactor Engineering and Safety, Department of Chemical Engineering, University of Leuven (KU Leuven), Leuven Chem&Tech, Leuven, Belgium

**Keywords:** Necrotrophy, oral bacteria, biofilms, hydrogen peroxide, killed cells

## Abstract

**Background:**

Bacteria respond to changes in their environment, such as nutrient depletion and antimicrobials exposure. Antimicrobials result not only in bacterial death, but also have a hand in determining species abundances and ecology of the oral biofilms. Proximity of dead bacterial cells to living ones is an important environmental change or stress factor. Dead bacteria represent high concentrations of nutrients, such as proteins, lipids, sugars, and nucleic acids. Living bacteria can use these biomasses as a nutrients source, which is termed necrotrophy.

**Aim:**

This study investigates the effect of exposing living oral bacteria (planktonic and biofilms) to their dead siblings after being killed by heat or hydrogen peroxide.

**Results:**

Tested bacterial species showed different responses towards the dead cells, depending on the mode of killing, the nutritional value of the culture media, and the the dead cells density. The multispecies oral biofilms showed different responses towards the supplementation of dead cells during biofilm development, while matured biofilms were more resilient.

**Conclusion:**

This study indicates that dead bacteria resulting from antiseptics use may imbalance the nutrient availability in the oral cavity, resulting in overgrowth of opportunistic species, and hence ecological changes in oral communities, or introducing new bacterial phenotypes.

## Introduction

In the oral cavity, heterogeneous microbial communities can be found. It is estimated that dental plaque contains 10^11^ cells/mL and saliva 10^9^ cells/mL [1]. The oral niche is a dynamic environment, with strong variations in environmental conditions e.g. pH, nutrient availability, mechanical disruption (brushing or flossing), antimicrobial exposure, different rates of salivary flow, and different salivary secreted compounds. These highly fluctuating conditions can stress bacterial cells, ultimately leading to bacterial death. In addition, the oral bacterial community faces daily multiple exposures to antimicrobials due to oral hygiene practices. Therefore, it can be assumed that bacterial death rates in the oral cavity are higher than in other extra-oral niches.

No doubt that using antiseptics in the oral care process should be regulated due to the risk of developing adaptation in the exposed oral microbial communities. Antiseptics adaptation is not the only problem, as cross antibiotic resistance was also reported [2]. Aside from reducing the total bacterial counts in the oral biofilms, antiseptics can lead to some ecological shifts in oral microbial communities, leaving behind more dysbiotic biofilms [3,4]. Nevertheless, antiseptic mouthrinses and toothpastes are still advised for specific groups of patients or subjects with high vulnerability to oral diseases [5]. Considering the recommended use of an antiseptic mouthrinse or toothpaste twice daily for these groups, with a killing efficiency of 99%, surviving oral bacteria will be exposed to dead bacteria at least twice a day. These surviving bacterial cells can use their dead relatives as a nutrient source [6,7]. Interactions between dead and living cells are not uncommon. This concept has been investigated for many Gram-negative and Gram-positive bacterial species. The response to dead cells can be diverse and is referred to by specific terminologies. ‘Necrotrophic growth’ refers to the use of dead bacterial biomass as a source of nutrients to grow on, while ‘necrovirulence’ indicates the increased virulence by the exposure to dead cells [8]. ‘Necrosignaling’ refers to the response of living bacterial cells to signals (e.g. proteins) released by dead cells [9]. Moreover, under particular stress conditions, a bacterial community can also induce ‘programmed cell death’ for part of the community to support the survival of the rest of the population [10].

Dead bacterial cells are able to induce changes in the exposed live bacteria independent of the cause of their death or the applied mode of killing, either physical, chemical or biological [7,11–13]. Physical (heat killing, autoclaving or UV irradiation), chemical (fumigation or using antimicrobials) or biological (bacteriophages-induced) methods of killing bacteria have different impacts on the target cells to be killed. In view of that, the dead bacterial cells are different enough to induce variable responses in the exposed live ones.

On top of affecting the exposed live cells, dead cells can also contribute to the biofilm development. The extracellular polymeric substances (EPS) in the matrix of the biofilms include proteins, polysaccharides, and extracellular DNA (eDNA). Although these proteins and polysaccharides can be produced and secreted by living cells, eDNA can only be obtained from lysed dead cells. Therefore, biofilm development is influenced by the release of eDNA from dead microbial cells [14].

Being frequently exposed to stress factors and antimicrobials, the oral microbiome may be one of the most significant communities to investigate the consequences of necrotrophy. The necrotrophic effects on oral biofilms after exposure to oral antiseptics are not known. Therefore, we hypothesize that oral bacterial communities exhibit necrotrophic activity towards dead bacteria that remain in the oral cavity after the use of oral antiseptics depending on the availability of other nutrients. This study therefore investigated the necrotrophic response of oral bacterial species and communities towards antiseptic-killed oral bacteria (using hydrogen peroxide) compared to heat-killed oral bacteria, with regard to inducing bacterial growth, community changes and ecological imbalances.

## Materials and methods

### 1. Bacterial strains, media, and culture conditions

*Prevotella intermedia* ATCC 25611, *Porphyromonas gingivalis* ATCC 33277, *Fusobacterium nucleatum* DSM 20482, and *Aggregatibacter actinomycetemcomitans* ATCC 43718 were included in this study as representatives of the periodontal pathogens. *Streptococcus mutans* ATCC 20523 and *Streptococcus sobrinus* ATCC 20742 were included as representatives of the cariogenic species. *Streptococcus gordonii* ATCC 49818, *Streptococcus oralis* DSM 20627, *Streptococcus sanguinis* LM 14657, *Streptococcus mitis* DSM 12643, *Streptococcus salivarius* TOVE-R, *Actinomyces viscosus* DSM 43327, *Actinomyces naeslundii* ATCC 51655, and *Veillonella parvula* DSM 2008 were included as oral commensals. All the species and strains were maintained on blood agar supplemented with hemin (5.0 mg/mL), menadione (1.0 mg/mL) and 5% sterile horse blood. Broth cultures were prepared in Brain Heart Infusion broth (BHI). L-Cysteine HCl at a concentration of 0.04% was added before autoclaving to the BHI used for growing the anaerobic periopathogens (*F. nucleatum*, *P. intermedia* and *P. gingivalis*) [15], while for the rest of the strains, only BHI without extra L-Cysteine HCl was used. BHI supplemented with 0.04% L-Cysteine HCl was noted as (BHIC). *F. nucleatum*, *P. intermedia*, *P. gingivalis*, *V. parvula*, *A. viscosus* and *A. naeslundii* were maintained under anaerobic conditions while the rest were maintained under 5% CO_2_.

Multispecies biofilm experiments were performed in Brain Heart Infusion 2 (BHI-2) broth. BHI-2 was prepared according to Herrero and co-workers by supplementing BHI with 2.5 g/L mucin, 1.0 g/L yeast extract, 0.1 g/L cysteine, 2.0 g/L sodium bicarbonate and 0.25% (v/v) glutamic acid [16]. A nutrient-free medium (NF) was prepared, containing sodium chloride 5 g/L and disodium hydrogen phosphate 2.5 g/L, which correspond to the inorganic fraction of BHI. Diluted BHI (DB) was prepared as one tenth the nutrients of the normal BHI. All the nutrient rich media (BHI, BHIC or BHI-2) are referred to as culture media (CM) in this study.

### 2. Bioreactor-derived multispecies biofilms

The multispecies communities were grown in a Biostat-B Twin bioreactor and the multispecies biofilms were grown on surface of standardized hydroxyapatite disks (HAD) under microaerophilic conditions as described earlier [4].

### 3. Preparation of dead bacteria

To prepare dead bacterial cells, overnight cultures of the selected species to kill were grown under the appropriate growth conditions. Afterwards, bacterial cells were collected by centrifugation at 6000 × g for 5 minutes.

For heat-killed (HK) bacteria, the collected cells were resuspended in either fresh CM or NF. Cells were then killed by incubating for 30 minutes at 95^◦^C. For peroxide-killed (PK) bacteria, the collected cells were resuspended in 3% H_2_O_2_ and incubated for 90 minutes at 37^◦^C with shaking at 250 rpm. After that, the cells were collected by centrifugation at 6000 × g for 5 minutes and the cells were washed twice; once with phosphate-buffered saline (PBS) supplemented with 1% hemin (to guarantee that any remaining H_2_O_2_ is consumed, and the effect is neutralized) and a second time with only PBS. After washing, cells were resuspended in either fresh CM or NF. Flow cytometry (FCM) was then used to check the possible damage of the cell membranes, which indicates the viability status of the cells, and measure the concentrations of the damaged cells in the suspensions. For more verification of the viability status and the efficacy of the killing procedures, 50 µL of each of the suspensions of the killed cells were plated on blood agar and incubated under the appropriate conditions for 48 hours in parallel with live cells as control for growth. No growth was observed in any of the killed suspensions.

### 4. Flow cytometry

Flow cytometry (FCM) analysis was performed using FACSVerse cytometer with a blue 488-nm laser and a red 640-nm laser (BD Biosciences, Belgium). The FACSVerse was operated with FACSFlow solution as a sheath fluid and the performance was verified by daily quality control using FACSuite CS&T beads. All measured samples were diluted beforehand in filter-sterilized PBS, to get the optimal detection rate for the machine during the measurement (200–2000 events/sec). This rate corresponds to a concentration of 10^5^ to 10^6^ cells/mL. A minimum of 10,000 cells per sample were measured to have accurate information enough for further analysis.

#### 4.1. FCM staining

All measurements were performed using a mixture of two fluorescent stains (live/dead staining technique), to have information not only on the total bacterial count but also on the fractions of intact (live) and membrane damaged (dead) bacterial cells. A mixture of a DNA stain, SYBR Green (SG), and a membrane integrity stain, propidium iodide (PI) was used and referred to as SGPI stain. The SGPI stain was added at a final concentration of 1% to the measured samples. Samples were then incubated in the dark after staining at 37^◦^C for 20 minutes.

#### 4.2. Sample acquisition

Forward scatter (FSC), side scatter (SSC), green fluorescence (FITC-A) and red fluorescence signals (PerCP-CY5.5-A) were measured for each sample after staining. The cells’ corresponding side scatter (SSC-A) was plotted against the forward scatter (FSC-A) to have information about the main bacterial cell characters, namely cell shape, size and dimensions (supplementary figure S1-a), which helps characterization of the bacterial species, while the red fluorescence (PerCP-CY5.5-A) was plotted against the green fluorescence (FITC-A) to have information about the viability status of the cells (supplementary figure S1-b). Quantification of both live and dead cells was possible afterwards using predefined cell gates.

#### 4.3. Gating cells in the FCM

Live bacterial cells and verified killed cells of each species as well as the 14-species biofilms were acquired separately with the FCM. Gates of the intact (live) and membrane damaged (dead) cells were assessed for each and used further for counting the cells according to their viability status (supplementary figures S1-c, d). A minimum of 10,000 cells per sample were measured to allow accurate quantification. The defined gates were used afterwards for quantifying the live and dead cells.

#### 4.4. FCM data analysis

The data obtained from the FCM for all the samples were denoised and analyzed using the *flowCore* and the *Phenoflow* packages in R (v4.0.3) [17].

#### 4.5. Phenotypic diversity analysis

After the FCM data were exported to R (v4.0.3) using the *flowCore* package, the *Phenoflow* package was used to characterize the phenotypic structure of the bacterial populations. Briefly, the live populations were gated, and the dead ones were excluded from the analysis. All the live populations then were subsampled to equal number of cells to avoid variations due to sample size differences. The phenotypic fingerprint was assessed for the required population, then it was used to calculate the beta diversity of the community according to Heyse and co-workers [18]. The beta diversity measures the change in the diversity of a certain species from one condition to another, which is then evaluated by a principal coordinate analysis (PCoA). The beta diversity is then indicated by a PCoA ordination plot, according to the Bray-Curtis dissimilarities between the fingerprints of different conditions.

### 5. Necrotrophy of single species

Overnight cultures of the tested species were measured for live cells concentrations using the FCM. Concentrations were adjusted to a final concentration of 7.7 Log_10_ events/mL in the experimental conditions, either using CM, DB or NF. Dead bacterial cells (HK or PK in CM or NF) were added to a calculated final concentration of either 7.7, 8.7, 9.4 or 9.7 Log_10_ events/mL, to achieve live : dead cells ratios of 1:1, 1:10, 1:50 or 1:100 respectively. For most of the experiments, a concentration of 9.7 Log_10_ events/mL of the dead cells was adjusted to achieve live : dead Log_10_ differences of 2 ± 0.2 (1.8:2.2 Log_10_ difference). Live cells inoculated in only CM or NF without the dead cells served as controls. The cultures were incubated for 24 hours in anaerobic or aerobic conditions (depending on the appropriate incubation conditions of the tested species) and then results were analyzed by FCM. All experiments were performed as four independent replicates.

### 6. Necrotrophy of multispecies oral biofilms

To test the effect of necrotrophy on biofilms, two approaches were investigated in this study: the effect of necrotrophy on established biofilms and the effect on developing biofilms. All experiments were performed as four independent replicates

#### 6.1. Necrotrophy in established biofilms

Biofilms were grown for 48 hours using bioreactor-derived multispecies communities on the surface of hydroxy apatite discs (HAD), under microaerophilic conditions as described earlier [4], either in CM or NF. After that, biofilms were moved to new well-plates, to be incubated with dead cells (HK or PK in CM or NF). Wells with only CM and NF served as control conditions for the biofilms. After 24 hours, biofilms were collected for FCM and v-qPCR analysis.

#### 6.2. Necrotrophy in developing biofilms

The effect of the presence of different dead bacterial cells on the biofilm development was tested according to the abovementioned setup, but biofilms were grown in presence of the killed cells. Biofilms grown in only CM of NF served as controls. The results were analyzed as abovementioned for the established biofilms.

### 7. Biofilm collection, DNA extractions and v-qPCR

Biofilms were detached from the HAD using 0.05% trypsin-EDTA as described earlier [4]. The biofilm pellets were then re-suspended in 500 µL PBS. for the FCM and v-qPCR analysis. DNA extractions and v-qPCR were performed as follows: 90 µL of the collected biofilms were treated with propidium monoazide xx (PMAxx) at a final concentration of 100 µM, incubated in the dark for 15 min, followed by photoactivation of the PMAxx for 30 min using Glo-Plate blue (Biotium, Hayward, CA, USA). Samples were then centrifuged for 10 min at 6000 × *g*. After that, DNA was extracted from the collected bacterial cells using QIAamp DNA Mini-kit (Qiagen, Hilden, Germany) following the manufacturer’s instructions. The qPCR assay was performed using a CFX96 Real-Time System (Bio-Rad, Hercules, CA, USA) and its associated software CFX Manager (version 3.1), using strain-specific primers and probes (supplementary table 1) according to Van Holm and co-workers [36].

### 8. Chemical oxygen demand assay

The chemical oxygen demand (COD) is the amount of oxygen needed to oxidize the organic matter in a sample. Therefore, it is considered an indication of the organic carbon content in a sample. The COD was assayed for HK and PK cells of the same species (*P. gingivalis*), to compare the carbon content, and hence the nutrient value, of both, and thus evaluating whether the killing method can have an effect on the nutrient value of the cells or not. COD was analyzed using the Nanocolor® kits NO.028 (Macherey-Nagel), according to the manufacturer instructions.

### 9. Statistical analysis

All statistical analyses were performed in R (v4.0.3). Normality of the residuals was assessed using Shapiro – Wilk test and a normal quantile plot, and equal variance for the different groups by Levene’s test. To test for significance for the differences between the growth conditions of the experiments and the ecological changes in the tested biofilms, Kruskal-Wallis test followed by Dunn multiple comparisons post-hoc analysis was used for the non-parametric data while ANOVA test with Tukey HSD multiple comparisons analysis was used in case of parametric data, with a confidence level of 95% for each. The phenotypic fingerprint based on FCM data was calculated using the *PhenoFlow* package in R (v4.0.3) as described earlier [17].

## Results

### 1. Necrotrophy with heat-killed (HK) cells

#### 1.1. Effect of HK *P. intermedia* cells on *P. intermedia*

**Figure 1.**
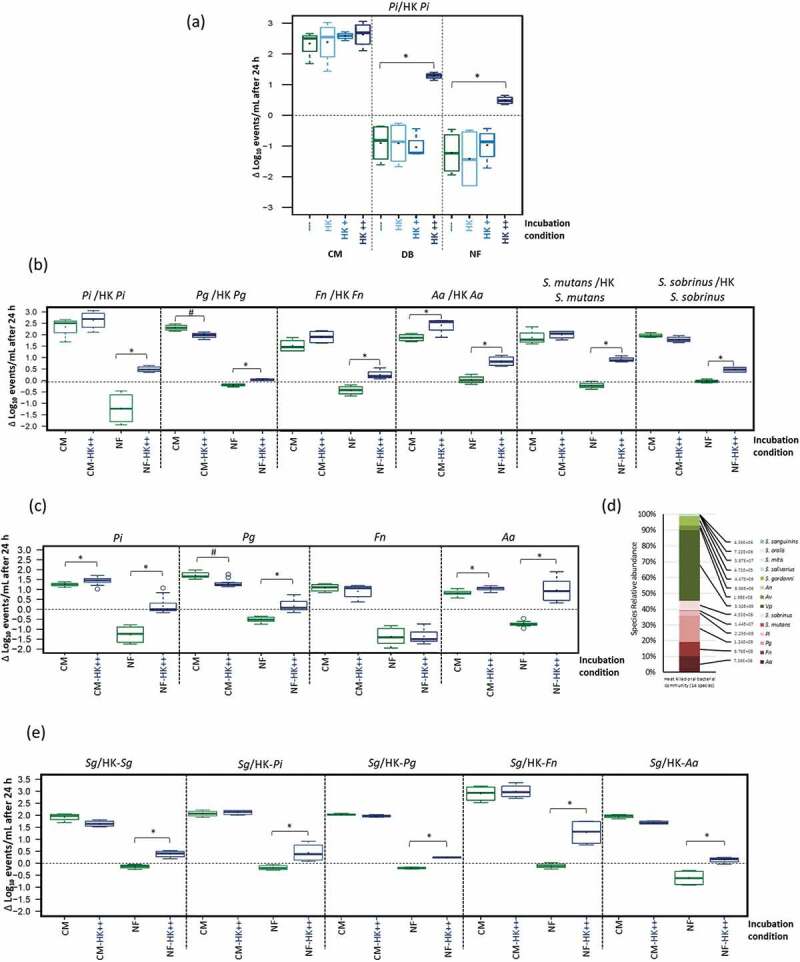
Necrotrophic response to heat-killed (HK) cells (expressed as the Log_10_ change in live bacteria after 24 hr incubation, and applied in different live : killed ratios): (a) response of *P. intermedia* (*Pi*) cells in different media (nutrient-rich medium (CM), diluted medium (DB) and nutrient-free medium (NF)) at different live : HK *P. intermedia* ratios (HK; 1:1, HK+; 1:10, and HK++; 1:100), (b); necrotrophic response of the tested periopathogens (*P. intermedia (Pi), P. gingivalis (Pg), F. nucleatum (Fn), and A. actinomycetemcomitans (Aa)*) and cariogenic species (*S. mutans* (*Sm*) and *S. sobrinus* (*Ss*)) towards HK cells of the same species (live : HK ratio of 1:100), in CM and NF, (c); necrotrophic response of the tested periopathogens towards HK cells of 14-species oral communities (live : HK ratio of 1:100), in CM and NF, (d); the composition and the numbers (Geq/mL) of each of the 14-species of the HK oral community used for necrotrophy experiment, (e); necrotrophic response of *S. gordonii* (*Sg*) towards HK cells of the same species and of the tested periopathogens (live : HK ratio of 1:100), in CM and NF. * Indicates significant necrotrophic growth/survival, and # indicates significant less growth/survival, compared to the corresponding control conditions.

Supplementing live *P. intermedia* in CM with different concentrations of HK *P. intermedia* resulted in a small (albeit non-significant) increase in growth (Figure 1a). In DB and NF, the number of viable *P. intermedia* cells decreased after 24 h incubation due to nutrient limitation. However, this nutrient limitation was completely countered when adding HK *P. intermedia* in a live : killed ratio of 1:100. This resulted, on average, in 2.18 and 1.7 Log_10_ events/mL more live *P*. *intermedia* than the corresponding control in DB and NF respectively.

#### 1.2. Necrotrophic response of periodontal and cariogenic pathogens towards HK relatives

The necrotrophic response of 3 other periopathogens (*P*. *gingivalis*, *F. nucleatum* and *A*. *actinomycetemcomitans*) and 2 cariogenic species (*S. mutans* and *S. sobrinus*) was investigated in CM and NF at a ratio of 1:100 (live : HK cells). In NF only, none of the species showed growth (Figure 1b). However, when HK cells were added, all tested species showed significantly better survival and even growth. In CM, there were no significant effects of HK cells on bacterial growth except for *A. actinomycetemcomitans* and *P. gingivalis*. HK *A. actinomycetemcomitans* caused a growth increase of an average of 0.54 Log_10_ events/mL (*p* < 0.005), while HK *P. gingivalis*, in contrast, inhibited living *P. gingivalis* with ≈0.34 Log_10_ events/mL (*p* < 0.05).

#### 1.3. Specificity of the necrotrophic activity

To check the specificity of the necrotrophic behaviour of an oral species towards a certain dead species, periopathogens in CM and NF were tested for their necrotrophic activity upon exposure to HK 14-species community in a live : HK ratio of 1:100 (Figure 1c). Based on community composition (shown in Figure 1d), it could be calculated that the live : HK ratio for each species was 1:10 for each of *P*. *intermedia*, *A*. *actinomycetemcomitans*, and *F. nucleatum*, and 1:20 for *P*. *gingivalis*. No necrotrophic response was observed for *F. nucleatum*, neither in CM nor NF (Figure 1c). In NF, *P. intermedia*, *P. gingivalis* and *A. actinomycetemcomitans* experienced a positive effect from the added HK community mix, either survival or growth. In CM, a bit of necrotrophic growth (≈0.2 Log_10_ events/mL), was observed for *P. intermedia* and *A. actinomycetemcomitans* (*p* < 0.001), which indicates no necrotrophic specificity towards a certain dead species. *P. gingivalis* in CM, on the contrary, displayed a small decrease (*p* < 0.001) in cell growth in the presence of HK community cells (≈0.42 Log_10_ events/mL).

#### 1.4. Necrotrophic activity of oral commensal species

The necrotrophic activity of *S. gordonii* (as a model organism for commensals) was investigated in CM and NF. The commensal was exposed to HK *S. gordonii*, *P*. *intermedia, P*. *gingivalis*, *F. nucleatum* and *A*. *actinomycetemcomitans* cells in a 1:100 live : killed ratio. In CM, adding the HK species did not affect *S. gordonii* growth. In NF, all species either increased growth or prevented the decrease in viable cells count that was observed in NF only (*p* < 0.05) (Figure 1e).

### 2. Necrotrophy on peroxide-killed (PK) cells

#### 2.1. Necrotrophic response of commensals with PK periopathogens

**Figure 2.**
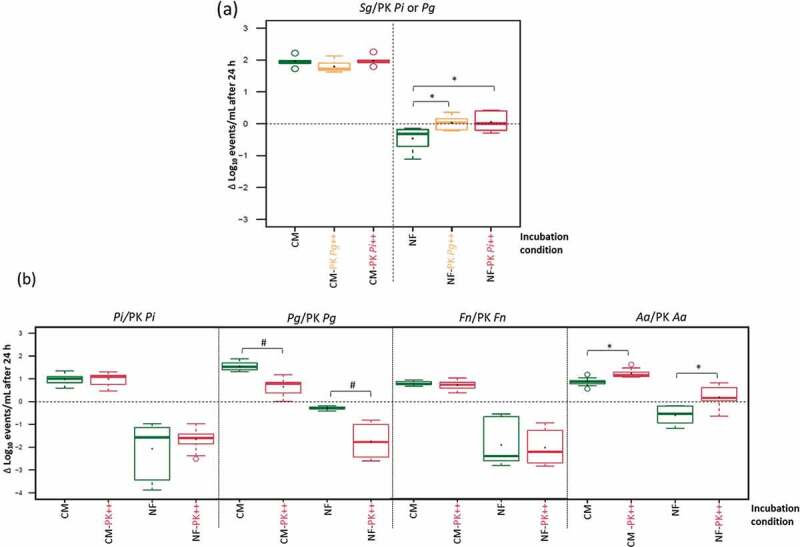
Necrotrophic response to peroxide-killed (PK) cells expressed as the Log_10_ change (live : PK ratio of 1:100): (a); necrotrophic response of *S. gordonii* (*Sg*) in different growth conditions, nutrient-rich medium (CM), and nutrient-free medium (NF), (b); necrotrophic response of the tested periopathogens towards PK cells of the same species (live : PK ratio of 1:100), in CM and NF. * Indicates significant necrotrophic growth/survival, and # indicates significant less growth/survival, compared to the corresponding control conditions.

Since *S. gordonii* can produce hydrogen peroxide, which can kill periopathogens, we exposed *S. gordonii* to peroxide-killed (PK) *P*. *intermedia* and *P*. *gingivalis* (Figure 2a). Supplementing CM with PK *P. intermedia* or *P*. *gingivalis* did not increase *S. gordonii* growth. In NF, *S. gordonii* showed some loss in viability which was prevented by adding PK *P. intermedia* or *P*. *gingivalis* at a ratio of 1:100 (*p* < 0.05), suggesting that that PK cells were used by *S. gordonii* as a nutrient source for survival.

#### 2.2. Necrotrophic response of periopathogens with PK relatives

Periopathogens were tested for possible necrotrophic behaviour toward PK cells from the same species (Figure 2b). Unexpected was the negative influence of PK *P. gingivalis* on the survival of living *P. gingivalis* itself in CM and NF. PK cells could further reduce the viable cell counts after 24 hr of exposure by an average of 0.91 and 1.46 Log_10_ events/mL in CM and NF respectively. No effect was observed when *P. intermedia* and *F. nucleatum* were subjected to PK cells of the same species, while a significant positive necrotrophic effect could be exerted by PK *A. actinomycetemcomitans* on live *A. actinomycetemcomitans* (≈0.38 Log_10_ events/mL more than the control).

### 3. Effect of PK *P. gingivalis* on oral bacteria

#### 3.1. Effect of PK *P. gingivalis* on *P. gingivalis* cells

**Figure 3.**
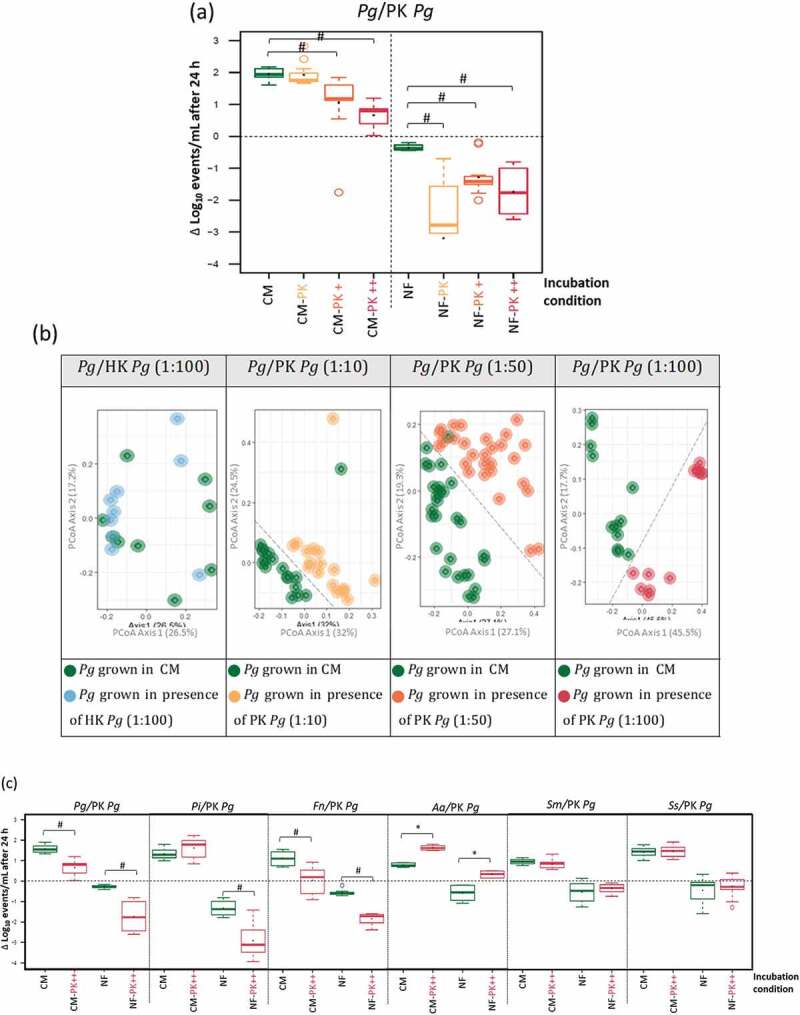
Effect of peroxide-killed *P. gingivalis* (PK *Pg*) on living *P. gingivalis* (*Pg*) cells at different live : PK *P. gingivalis* cells ratios (PK; 1:10, PK+; 1:50 and PK++; 1:100): (a); growth necrotrophic response of live cells to PK *P. gingivalis* (PK *Pg*) cells, represented as the change in the Log_10_ values of the live cells than the starting values (Δ Log_10_) (b); beta diversity of live *P. gingivalis* (*Pg*) cells grown in presence of heat-killed *P. gingivalis* cells (HK *Pg*) or different concentrations of PK *P. gingivalis* (PK *Pg*) cells, compared to corresponding live *P. gingivalis* cells grown in only CM. Each circle represents the phenotype of living *P. gingivalis* cells of an independent replicate, (c); necrotrophic response of periopathogens and cariogenic species towards PK *P. gingivalis* cells (live : PK ratio of 1:100), in CM and NF. * Indicates significant necrotrophic growth/survival, and # indicates significant less growth/survival, compared to the corresponding control conditions.

The observed inhibitory effect of PK *P*. *gingivalis* on the growth and survival of living *P*. *gingivalis* was further investigated by supplying living *P*. *gingivalis* with different live : PK *P*. *gingivalis* cells ratios (1:10, 1:50 and 1: 100) in CM and NF (Figure 3a). A negative effect was observed on growth in CM at 1:50 and 1:100 ratios, and on survival in NF media at all tested ratios (*p* < 0.05).

#### 3.2. Phenotypic changes in *P. gingivalis* grown while exposed to HK *P. gingivalis* and different concentrations of PK *P. gingivalis*

Phenotypes of living *P*. *gingivalis* grown in CM in presence of HK/PK *P*. *gingivalis* were analysed in comparison to *P*. *gingivalis* grown in CM only, by comparing the beta diversity for each community. As shown in Figure 3b, clear diversity between the two populations was observed when *P*. *gingivalis* was grown in presence of PK cells. This effect was not observed for HK *P*. *gingivalis*.

#### 3.3. Effect of PK *P. gingivalis* on periopathogens and cariogenic species

The inhibitory effect of PK *P*. *gingivalis* towards other periopathogens and cariogenic species was investigated in CM and NF (Figure 3c). A significant inhibitory effect was observed for PK *P*. *gingivalis* cells on *P*. *intermedia* and *F. nucleatum* as well, but not on *S. mutans* or *S. sobrinus*. In contrast, *A*. *actinomycetemcomitans* showed not only a better survival in NF supplemented with PK *P*. *gingivalis*, but also a significant increase in growth in CM supplemented with PK *P*. *gingivalis* (≈0.91 & 0.84 Log_10_ events/mL more cells than corresponding control respectively).

### 4. Nutritional value of PK versus HK cells

Due to the different necrotrophic effects on the living cells, the nutritional value of both types of killed cells was investigated. The organic carbon content (chemical oxygen demand (COD)) was measured for both HK and PK *P*. *gingivalis* cells as a guide for the HK and PK cells. PK cells showed lower COD, than HK cells. PK *P*. *gingivalis* had COD value of 0.4 g/L vs 1.1 g/L for HK *Pg*. This then can elucidate the less to no contribution of PK cells of *P*. *gingivalis* to the necrotrophic survival of the living cells in NF media.

### 5. Necrotrophic responses of oral biofilms to HK and PK cells

The effect of necrotrophy was tested in oral biofilms in two approaches; during biofilm development and after the biofilm was developed. Due to the inhibition of PK *P*. *gingivalis* towards some periopathogens, the effect of PK *P*. *gingivalis* and PK *P*. *intermedia* on multi-species biofilms in CM and NF was tested. HK cells served as controls.

#### 5.1. Necrotrophy in developing biofilms

**Figure 4.**
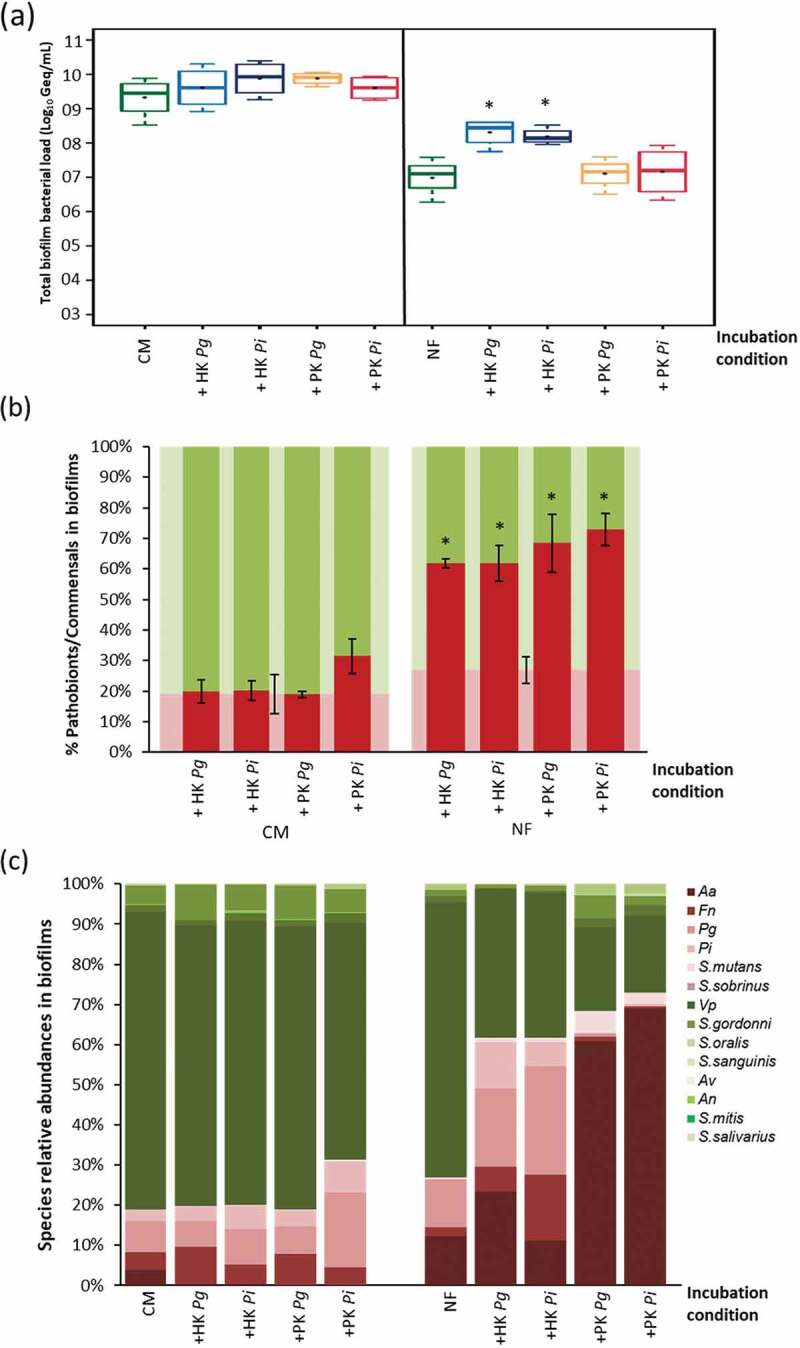
Effect of necrotrophic activity of oral bacteria towards differently killed oral species on biofilms formation: (a); total biofilm bacterial load represented as Log_10_ Geq/mL in presence/absence of heat-/peroxide-killed cells of *P. gingivalis*/*P. intermedia* (live : PK ratio of 1:100) in CM and NF, * indicates significantly more biofilm formation relative to the corresponding control condition without the supplied killed cells, (b); ecological changes, expressed as percentages of commensals (green) and pathobionts (red) incidence in the biofilm, associated with adding the killed cells to the biofilms during development, error bars represent the standard error of pathobionts percentage (*n* = 4), * indicates significantly more dysbiosis in the biofilms relative to the corresponding control condition (showed in the background of the tested conditions) without the supplied killed cells, (c); biofilms composition and species relative abundances changes (represented in %) associated with adding the killed cells to the biofilms during development.

Supplementation of killed cells to a developing biofilm had no significant effect on the total cell number after 24 hr in CM (Figure 4a). In NF, HK *P. gingivalis* and *P. intermedia* increased the final bacterial numbers in the biofilms (*p* < 0.05), whereas the PK cells had no significant effect. When looking at the ecological changes, all conditions in NF resulted in an altered ecology (Figure 4b). More precisely, the percentage of the pathobionts increased from 27% (for NF) up to ≈62% (for HK) and to ≈70% (for PK), depending on the mode of killing. Noteworthy, this ecological shift was not observed in CM. The 3 periopathogens (*F. nucleatum, P*. *gingivalis* and *P*. *intermedia*) numbers were higher (*p* < 0.05) in presence of HK *P*. *gingivalis* or *P*. *intermedia* in NF, while *A*. *actinomycetemcomitans* had higher numbers with only HK *P*. *gingivalis* in NF. In contrast, adding PK cells to NF during biofilm development increased the abundance of only *A*. *actinomycetemcomitans* (*p* < 0.05) to ≈61% and 69%, with PK *P*. *gingivalis* and PK *P*. *intermedia* respectively, versus ≈12% abundance in the NF control (Figure 4c). *A*. *actinomycetemcomitans* showed better growth in presence of the PK cells in NF (6.83 ± 0.48, 7 ± 0.67 Log_10_ Geq/mL in NF with PK *P*. *gingivalis* and *P*. *intermedia* respectively VS 6.05 ± 0.51 Log_10_ Geq/mL in NF only). On the other hand, the added PK *P*. *gingivalis* and PK *P*. *intermedia* cells reduced the abundance of *P*. *gingivalis* (0-1%) versus 12% in the NF-grown biofilms, due to a significant reduction of *P*. *gingivalis* numbers in presence of the PK cells (≈1.21, 2.03 Log_10_ Geq/mL decrease in NF with PK *P*. *gingivalis* and *P*. *intermedia* respectively than in NF only). Also, significantly lower numbers of *F. nucleatum* were detected in presence of PK *P*. *intermedia* (≈1.3 Log_10_ Geq/mL less than the NF control).

#### 5.2. Necrotrophy in established biofilms

**Figure 5.**
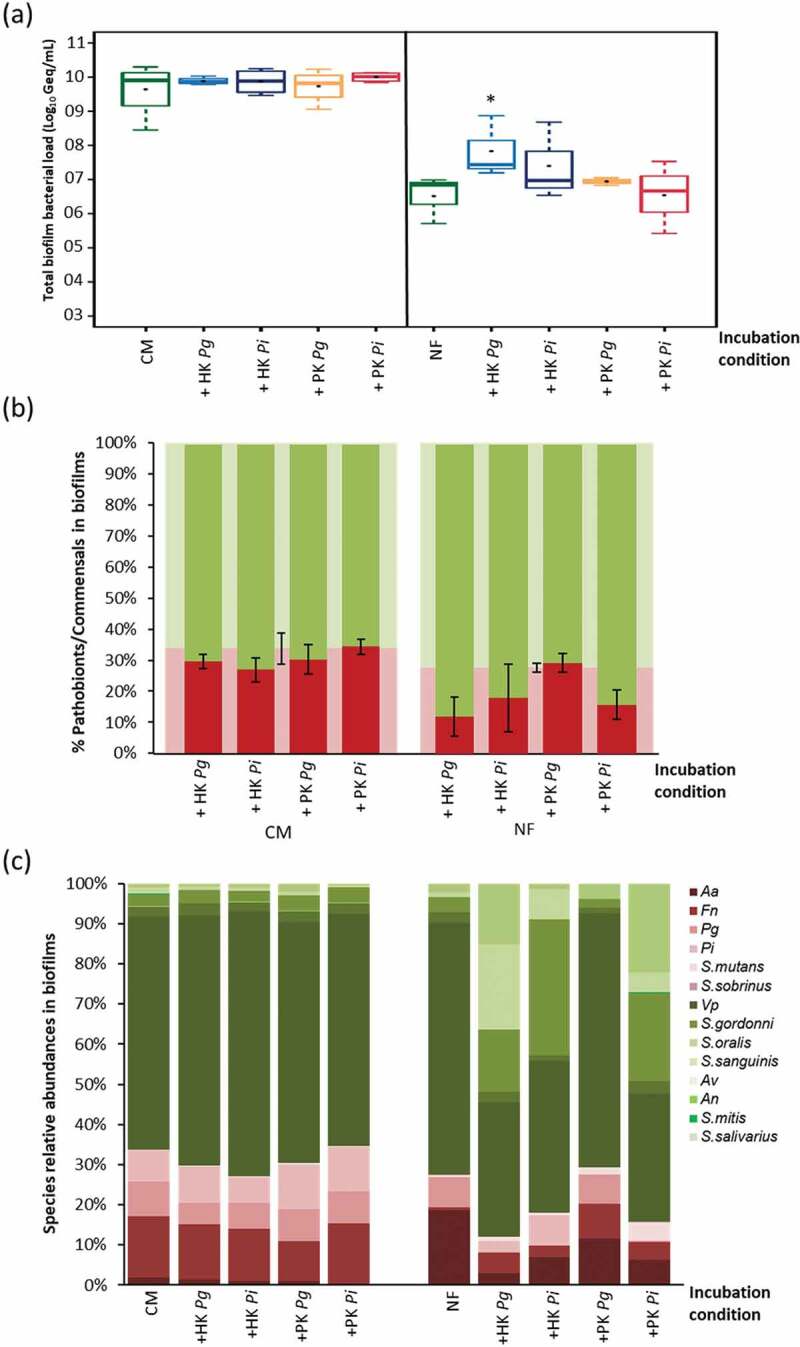
Effect of necrotrophic activity of oral bacteria in biofilms towards differently killed oral species: (a); total biofilms bacterial load represented in Log_10_ Geq/mL in presence/absence of heat-/peroxide-killed cells of *P. gingivalis*/*P. intermedia* (live : killed ratio of 1:100) in CM and NF, * indicates significantly more biofilm formation relative to the corresponding control condition without the supplied killed cells, (b); ecological changes, expressed as percentages of commensals (green) and pathobionts (red) incidence in the biofilm, associated with incubating the formed biofilms with the killed cells, error bars represent the standard error of pathobionts percentage (*n* = 4), no significant ecological shifts in the biofilms relative to the corresponding control condition (shown in the background of the tested conditions) without the supplied killed cells, (c); biofilms composition and species relative abundances changes (represented in %) associated with incubating the formed biofilms with killed cells.

For established biofilms, first grown for 48 hr and afterwards exposed to HK and the PK cells, the observations were different. No significant changes in biofilms quantities were observed, neither in CM nor in NF, except with HK *P*. *gingivalis* in NF (Figure 5a). However, the ecological response in the presence of killed cells was different than in the developing biofilms (Figure 5b). The ecological balance did not significantly change, with ≈29% incidence of pathobionts in the PK *P. gingivalis* exposed biofilms versus ≈28% in the NF control biofilms. The same also was observed for all the NF experimental conditions. Moreover, in CM, the community composition, and species abundances did not change in any of the tested conditions (Figure 5c). On the other hand, in NF, some changes in the community composition and species abundances were noted, with mainly a shift from *V. parvula* dominance to more streptococcal abundance for the commensals with no significant changes to the pathobionts/commensals ratio.

## Discussion

This study investigates the phenomenon of necrotrophy, which was first described in the field of oral microbiology by Herrero and co-workers [8] but remains rather unexplored to date. Exposure of living cells to dead cells is common in the oral cavity due to the use of antiseptic oral hygiene products for improving oral health. We hypothesize that the mode by which dead bacteria were killed can have an impact on necrotrophic response of live cells towards them. Different modes of killing bacteria include physical (e.g. UV, heat, or autoclaving) and chemical (e.g. antiseptics, disinfectants, or fumigation) methods. It is reported that dead cells can be used as a nutrient source in case of nutrient depletion, to help in the survival of cohabitating bacteria [19]. However, the effect of dead cells can go beyond just merely providing nutrients for the survivors and can affect the virulence of the necrotrophic species or the biofilms species abundances and hence biofilms ecology and homeostasis [8,13].

In the present study, two methods of bacterial killing were approached: heat killing (physical killing), and antiseptic killing (chemical killing) using hydrogen peroxide (H_2_O_2_). Exposure of bacterial cells to heat causes damage in the bacterial membranes (inner and outer), and hence disrupting the permeability barrier, leading to leakage of periplasmic proteins as well as the intracellular components. The heat exposure also results in denaturation of proteins, DNA and RNA degradation, and enzymes inactivation [20]. On the other hand, killing the bacterial cells using H_2_O_2_ causes oxidative damage to cell membranes, lipids, DNA, proteins, and amino acids [21].

Starting with the heat-killed (HK) bacterial cells, necrotrophic activity of *P*. *intermedia* was assessed in a nutrient-rich culture medium (CM), a tenfold diluted culture medium (DB), and a nutrient-free medium (NF). HK *P*. *intermedia* cells were used as representatives of physically-killed cells, with no added chemicals. They were added at a live : HK cell ratio of 1:1, 1:10 and 1: 100. It was assumed that the dead cells could act as extra nutrient sources in CM and DB, and they were the only nutrient source in NF. Significant effects were observed when the live : HK cells ratio was 1:100 for all the tested species. The dose-dependent necrotrophic effect was similar to the observations of Temmerman and co-workers, who reported necrotrophic growth of *Legionella pneumophila* when 100 dead cells were available for 1 live cell [7].

Other periopathogens and cariogenic species were tested for their necrotrophic activity. All the tested live species (except *F. nucleatum*) showed necrotrophic behaviour and were able to use the HK cells of a 14-species community to grow or survive. The HK 14 species all together were provided at a 1:100 live : HK cells ratio, but each species from the killed community was provided at a maximum live : HK cells ratio of 1:20. Earlier studies reported species-specific necrotrophy only at a 1:100 live : killed cells ratio for *P. intermedia*, *P*. *gingivalis*, *L. pneumophila*, and drinking water bacterial communities [7,8,11]. However, unlike the abovementioned studies, the killed cells in this study were provided to the live ones as a mixed-species community with a live : HK cells ratio below 20 at a species level. This indicates that living cells can use nutrients coming from different species simultaneously and the live: HK ratio should be seen at a community level. This is important in the oral cavity which comprises multispecies communities where there are no barriers between the cohabitant species.

In contrast to the other tested species, *A*. *actinomycetemcomitans* showed an increase in growth in CM-HK. This can be explained by the fastidious nature of *A*. *actinomycetemcomitans*, and its complex nutrient requirements, which makes it grow better in presence of extra nutritional sources [22,23].

However, the observed decreased growth of *P*. *gingivalis* in CM-HK (*P*. *gingivalis* or the 14-species community), but not in NF-HK, confirms that the response of live cells towards dead cells not only depends on the availability of other nutrients [13]. Similarly, *E. coli* grown in nutrient-rich media supplemented with dead cells showed less growth compared to cultures without dead cells, while growing better in nutrient-deficient media supplemented with the killed cells than without supplementation [13]. This observation can be explained by quorum sensing. Dead cells can contribute to the quorum sensing signals and subsequently interfere with the density-dependent growth response [24,25]. According to Allocati and co-workers, the accumulation of quorum sensing molecules can activate different programmed cell death pathways in bacterial cells to control the bacterial load [10]. Dead cell signalling (necrosignaling) is not uncommon among both Gram-negative and Gram-positive bacteria [9]. Cross-species responses to necrosignaling molecules have been reported between *E. coli* and *Salmonella*, and *P. aeruginosa*, and *B. subtilis*, leading to high population density-induced cell death, which indicates possible non-species specificity of necrosignaling, as long as the live cells have receptors for the necrosignaling molecules [9,26]. However, cell density could not explain the inhibitory effect of dead cells on living *E. coli* because the inhibitory effect was independent of the starting population density [13].

As the oral microbial community encompass both pathogens and commensals, cohabitating the same niches, the necrotrophic activity of oral streptococci, one of the most abundant commensal species in the oral microbiome, was investigated. Oral streptococci produce hydrogen peroxide in the oral cavity by which they control the growth of periopathogens [16,27]. *S. gordonii* showed necrotrophic activity in NF-HK of *S. gordonii, P*. *intermedia, P*. *gingivalis, F. nucleatum* and *A*. *actinomycetemcomitans*. These observations expand on the reported necrotrophic activity of *S. gordonii* towards killed *P*. *gingivalis* in 14-species communities [8]. They also indicate a possible necrotrophic competition for nutrients, available in the form of dead cells, between pathobiont and commensal species.

While heat-killed cells were mainly used to demonstrate the existence of necrotrophic effects, this study also investigated a more realistic condition, in which cells were killed using hydrogen peroxide (PK cells), which is known to be produced by streptococci in the oral cavity. Even though *S. gordonii* could better survive in NF-PK of *P*. *intermedia* and *P*. *gingivalis*, these PK cells did not affect the survival of living *P*. *intermedia* and *F. nucleatum* in NF, and even reduced the survival of *P. gingivalis*. PK *P. gingivalis* also reduced the survival of *P. intermedia* and *F. nucleatum* in NF and the growth of *F. nucleatum* in CM. On the other hand, *A*. *actinomycetemcomitans* could survive better in NF-PK of *A*. *actinomycetemcomitans* cells and showed higher capability to recycle the nutrient from PK *P. gingivalis* cells in CM and NF as well. This points out the strong necrotrophic activity of *A*. *actinomycetemcomitans*.

The different killing methods used in this study can explain the different responses to the dead cells. The phenotypic diversity and variation observed when *P. gingivalis* was grown with PK (and not with HK) *P. gingivalis* indicates a stress exposure. Under stress conditions, the gene expression profile of bacteria changes [28]. Variations in gene expression among the bacterial cells of the same species are one of the main reasons behind phenotypic heterogenicity [18]. Smakman and co-workers also reported that live cells can respond in a different way to killed cells depending on the way they were killed [13]. Also, killing bacterial cells with hydrogen peroxide leads to damage to the cellular proteins and lipids, and by this, they cannot add enough nutrient value to the media [21,29]. This was confirmed when the chemical oxygen demand (COD) was measured for both the HK and PK cells of the same bacterial species (*P. gingivalis*). The COD is used here for the quantification of the organic matter, which is the amount of nutrients provided from bacteria in the NF in this case. The COD of the HK *P. gingivalis* was 2.7 times more than that of the PK *P. gingivalis* for the same concentration of the killed cells.

Since oral communities exist in the form of biofilms on different oral surfaces, the necrotrophic response of developing (growing) and developed (established) biofilms was investigated. PK cells (as representative of antiseptic-killed cells) were tested versus HK cells (control physical killing of cells). The reported increased biofilm formation in NF-HK in this study can be explained by the fact that the HK cells release eDNA after being killed, which then contributes to the formation and stability of biofilms. The contribution of eDNA to biofilm formation via promoting bacterial adhesion to surfaces is well-reported [10,30,31]. Since hydrogen peroxide can cause oxidative DNA damage, PK cells did not promote biofilm formation [21,29,32]. However, *A*. *actinomycetemcomitans* abundances were significantly elevated in biofilms in NF-PK compared to control biofilms. PK cells improved *A*. *actinomycetemcomitans* survival in biofilms, but not the other periopathogens, which caused *A*. *actinomycetemcomitans* dominance in the formed biofilms. Similarly, subjects with periodontal diseases were reported to have a higher specific oxidation index and *A*. *actinomycetemcomitans* was reported to be the most prevalent bacteria in these periodontitis patients [33,34]. Thus, the presence of the PK cells during biofilm formation could improve the growth of *A*. *actinomycetemcomitans*. On the other hand, in presence of HK cells in NF, all the living bacterial cells were able to use the killed cells as nutrient sources and then all species had equal chances of getting nutrients and hence survival. Consequently, the total biofilm amount was significantly higher in NF-HK than in NF alone. In presence of HK cells in a nutrient poor media, the competition between all the bacterial species for nutrients results in ecological changes in favour of the pathobionts. Similarly, necrotrophic growth was reported earlier to be a potential virulence factor in oral pathogens [8].

HK cells, and not PK cells, caused some increase in biofilm quantity when added to NF after the biofilm formation as well. This ability of bacterial communities to use the dead cells to survive and grow was also reported in the past in different fields [10]. Noteworthy, in the established biofilms, neither *P. intermedia, P. gingivalis* nor *F. nucleatum* showed a significant reduction in numbers in presence of PK cells. This contrasts what was observed in developing biofilms. The observation highlights an important advantage behind cohabitating in a multicellular multispecies structured biofilms, which is getting protection and support [10]. *P. intermedia*, *P. gingivalis* and *F. nucleatum* could tolerate exposure to the PK cells, while cohabitating in a biofilm structure, more than what was observed in axenic cultures or biofilms during initial development. The stress-protection effect of cohabitating in a biofilm structure is well reported for oral species in general, and for *P. gingivalis* in specific [35]. So, the ecology of the biofilms was somehow stable regardless of the type of added cells.

All the observations from this study support the hypothesis that necrotrophic growth exists among oral bacterial species, commensals and pathobionts. The necrotrophic activity of the pathobionts can then be considered as an additional virulence factor in case of nutrient deficiency-related stress. Findings from this study also indicated that the mode of killing the bacteria (source of the nutrients) may act as a selective factor for the survival of some periopathogens, and thus other modes of bacterial killing (e.g. chlorhexidine or cetylpyridinium chloride killing) has to be taken into account for further investigations. Furthermore, this study was conducted *in vitro* using laboratory-grown strains, and further *ex vivo* inocula from different subjects should be considered for growing microcosm biofilms for future studies.

## Conclusion

Different responses of living bacterial cells to dead cells among different tested oral species help in the selective growth of some species. This depends on how the dead cells were killed, nutrient availability and their quantity. When nutrients are limited, HK cells increased the growth of living cells while the PK cells did not. The exact reason behind the inhibitory effect of the PK cells, in general, and PK *P. gingivalis*, in specific, is, however, not known and beyond the scope of this study.

## Supplementary Material

Supplemental Material

Supplemental Material

Supplemental Material
