# Supplementary material for: Mode of killing determines the necrotrophic response of oral bacteria
Source: J Oral Microbiol. 2023 Mar 6;15(1):2184930. doi: 10.1080/20002297.2023.2184930 (PMC10013485; doi:10.1080/20002297.2023.2184930)
Supplement: Supplemental Material [file ZJOM_A_2184930_SM9360.zip › Supplementary files/Supplementary information.docx]

## Supplementary figure 1

Flowcytometry analysis of the acquired bacterial cells. (a); The acquired cells’ side scatter (SSC-A) plotted against the forward scatter (FSC-A), a gate was then drawn and assigned to the corresponding cells. (b); The red fluorescence (PerCP-CY5.5-A) of a random sample plotted against its green fluorescence (FITC-A) to show the intact and damaged cells in the sample. (c); control live bacterial cells and (d); control killed bacterial cells acquired separately with the FCM. Gates of the intact (c) and membrane damaged (d) cells were assessed for each species and used further for quantifying the cells according to their viability status.

# Supplementary information

**Supplementary table 1:** Primers & qPCR conditions for v-qPCR. qPCR was performed with a CFX96 real-time system (Bio-Rad, Hercules, CA, USA). The reactions mixtures consisted of 12.5 mL of Takyon Rox probe master mix dTTP blue (Eurogentec, Seraing, Belgium), 1 mL of each primer (IDT, Haasrode, Belgium) and probe (all DD probes, 5'-FAM [6-carboxyfluorescein] and 3'-TAMRA [6-carboxytetramethylrhodamine]; Eurogentec, Seraing, Belgium) and 4.5 mL of Milli-Q water. Cycle conditions were as follows: an initial step at 50°C for 2 min and 95°C for 10 min, followed by 45 cycles of 95°C for 15 s and 60°C for 1 min.

| **Species** | **Primers & Probe (Final concentrations)** | | **Amplicon length (bp)** | **Target** |
| --- | --- | --- | --- | --- |
| *Aggregatibacter actinomycetemcomitans* | Forward | CGG TGT CGA TTT GGG GAT TGG(300 nM) | 237 | 16S rRNA gene |
|  | Reverse | TGC AGC ACC TGT CTC AAA GC (300 nM) |  |  |
|  | Probe | AGA ACT CAG AGA TGG GTT TGT GCC TTA GGG (100 nM) |  |  |
| *Prevotella intermedia* | Forward | TGT GCC CYT TTG CAT TTA CCC TTC(300 nM) | 216 | 16S rRNA gene |
|  | Reverse | CAC CAT GAA TTC CGC ATA CG (900 nM) |  |  |
|  | Probe | TGG CGG ACT TGA GTG CAC GC (200 nM) |  |  |
| *Porphyromonas gingivalis* | Forward | CCG TAA GAA TAA GCA TCG GCT AAC TC (300 nM) | 195 | 16S rRNA gene |
|  | Reverse | CAC GAA TTC CGC CTG C (300 nM) |  |  |
|  | Probe | CAC TGA ACT CAA GCC CGG CAG TTT CAA (100 nM) |  |  |
| *Fusobacterium nucleatum* | Forward | GGA TTT ATT GGG CGT AAA GC (300 nM) | 191 | 16S rRNA gene |
|  | Reverse | ATC TGT CCA GTA AGC TGG CTT CC (300 nM) |  |  |
|  | Probe | CTC TAC ACT TGT AGT TCC G (300 nM) |  |  |
| *Streptococcus mutans* | Forward | GCC TAC AGC TCA GAG ATG CTA TTC T (900 nM) | 114 | gtfB gene |
|  | Reverse | GCC ATA CAC CAC TCA TGA ATT GA (900 nM) |  |  |
|  | Probe | TGG AAA TGA CGG TCG CCG TTA TGA A (100 nM) |  |  |
| *Streptococcus sobrinus* | Forward | AAA TAC GGC CAG TGC CAA AG (200 nM) | 165 | gtfT gene |
|  | Reverse | CCA GCC TGA GAT TCA GCT TGT (200 nM) |  |  |
|  | Probe | CCT GCT CCA GCG ACA AAG GCA GC (250 nM) |  |  |
| *Actinomyces naeslundii* | Forward | TCG AAA CTC AGC AAG TAG CCG (200 nM) | 96 | gene encoding unknown protein |
|  | Reverse | AGA GGA GGG CCA CAA AAG AAA (200 nM) |  |  |
|  | Probe | GGG TAC TCT AGT CCA AAC TGG CGG ATA GCG (100 nM) |  |  |
| *Actinomyces viscosus* | Forward | GTG AAG GAG CCA GCT TGC TGG TTC TG (200 nM) | 155 | 16S rRNA gene |
|  | Reverse | CGG AAC AAA CCT TTC CCA GGC (200 nM) |  |  |
|  | Probe | ATG AGT GGC GAA CGG GTG AGT AAC (125 nM) |  |  |
| *Veillonella parvula* | Forward | GAC GAA AGT CTG ACG GAG CA (200 nM) | 171 | 16S rRNA gene |
|  | Reverse | TGC CAC CTA CGT ATT ACC GC (200 nM) |  |  |
|  | Probe | AGC TCT GTT AAT CGG GAC GAA AGG C (125 nM) |  |  |
| *Streptococcus oralis* | Forward | ACC AGC AGA TAC GAA AGA AGC AT (400 nM) | 229 | *gtfR* gene |
|  | Reverse | AGG TTC GGG CAA GCG ATC TTT CT (400 nM) |  |  |
|  | Probe | AAG GCT GCT GTT GCT GAA GAA GT (100 nM) |  |  |
| *Streptococcus sanguinis* | Forward | CAA AAT TGT TGC AAA TCC AAA GG (600 nM) | 75 | *gtfP* gene |
|  | Reverse | GCT ATC GCT CCC TGT CTT TGA (600 nM) |  |  |
|  | Probe | AAA GAA AGA TCG CTT GCC AGA ACC GG (100 nM) |  |  |
| *Streptococcus gordonii* | Forward | GAA GAA CTG GGT AGC GAT TGC T (400 nM) | 262 | *gtfG* gene |
|  | Reverse | GTT AGC TGT TGG ATT GGT TGC C (400 nM) |  |  |
|  | Probe | AGA ACA GTC CGC TGT TCA GAG CAA (100 nM) |  |  |
| *Streptococcus mitis* | Forward | GGC TCG TAG TCT GGA GAT GG (600 nM) | 133 | 16S rRNA gene |
|  | Reverse | TAG GTC GTC GTC CCA AGG AA (600 nM) |  |  |
|  | Probe | CGA AGA GCA CCA ATA GCA CCT CCC (140 nM) |  |  |
| *Streptococcus salivarius* | Forward | GAC GAT GAC TGT CAA CTT GAC AC (400 nM) | 247 | *Dextranase* gene |
|  | Reverse | ACC GTA ACG TGG GAA AAC TG (400 nM) |  |  |
|  | Probe | GTA GCG TCA GAG TGG TTG AC (100 nM) |  |  |
