# Supplementary figures and images for: Mode of killing determines the necrotrophic response of oral bacteria
Source: J Oral Microbiol. 2023 Mar 6;15(1):2184930. doi: 10.1080/20002297.2023.2184930 (PMC10013485; doi:10.1080/20002297.2023.2184930)

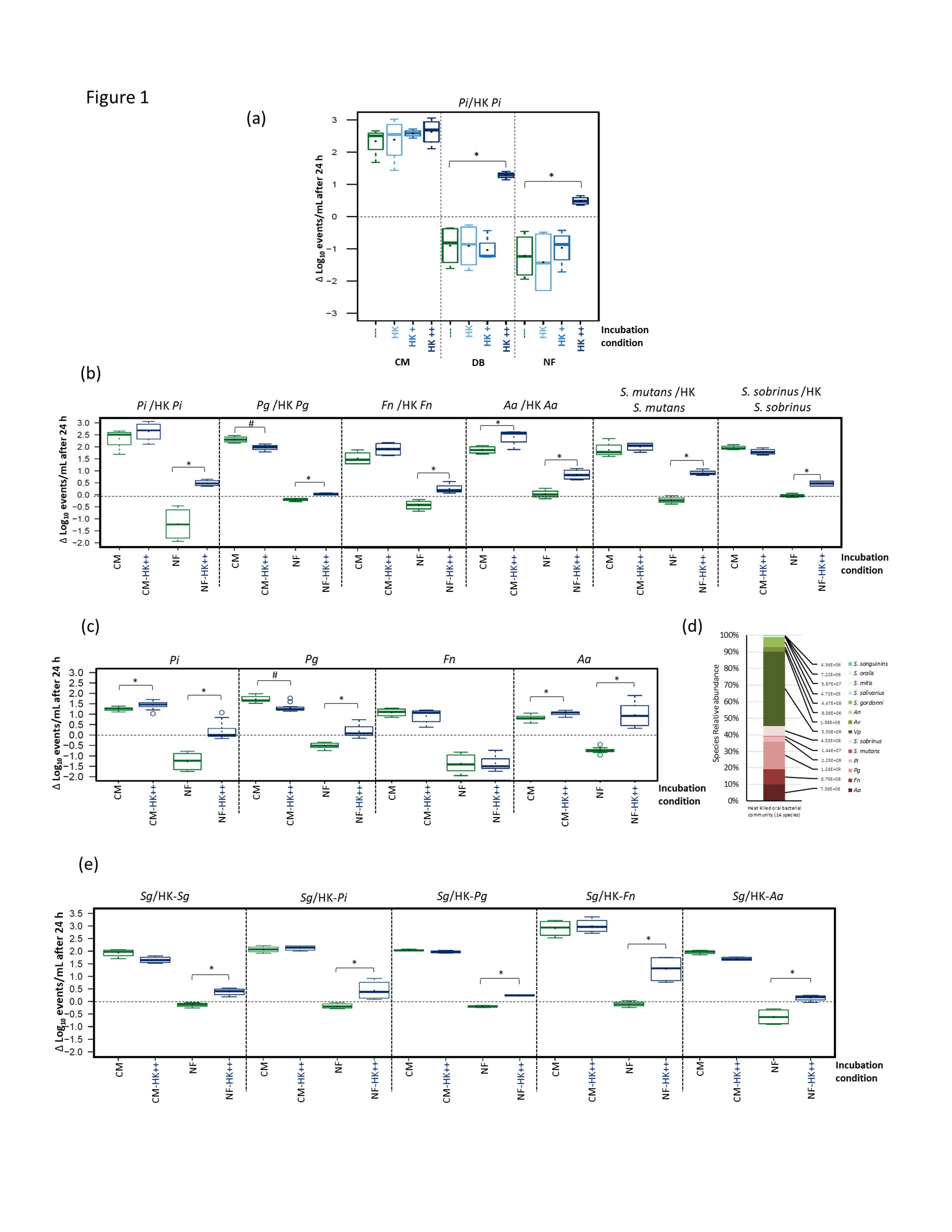

Supplement: Supplemental Material [file ZJOM_A_2184930_SM9360.zip › Supplementary files/Supplementary Figure 1.jpg]
